# The Yin & Yang of Data Management - Four intractable contradictions and their harmonious resolutions

**DOI:** 10.23889/ijpds.v1i1.366

**Published:** 2017-04-19

**Authors:** Alex Hacker

**Affiliations:** 1 The Nuffield Department of Population Health

## Objectives

In our long-term prospective cohort study of over 500,000 adults, participants are actively followed-up through established morbidity and mortality registries, and by linkage to the national health insurance system. We also administer regular questionnaires; take physical measurements; and gather genetic, metabolomics, and even meteorological data.

Importing, integrating and distributing such a large and diverse dataset presents practical challenges, but also more fundamental ones. When individually reasonable data management requirements come into conflict, can a solution be found that satisfies both without compromising either? In this session I present, with examples, four such conflicts and the ways in which we harmonise and resolve them.

## Approach & Results

### Gather perfect data / Handle imperfect data

A data manager does not want to allow erroneous data into their database, but nor do they want to discard data that are imperfect but still meaningful. The approach that we take is to implement automatic validation at the point of entry, tailored to the data source in question.

### Fix data issues / Don't make assumptions

Ideally every data issue that is detected should be fixed or flagged, but correction is not always possible and it's rarely clear where to draw the line between error and outlier. We address this via comprehensive data documentation, empowering each analyst to identify, assess and handle the values that might be problematic for them.

### Be flexible / Be consistent

Every researcher has different data requirements, definitions and exclusions. Data management must support this without needless duplication of effort, or leaving everyone working on incompatible datasets. Our solution is centralised distribution: a single core database, centrally maintained and updated, from which all analyst's datasets are derived, and into which the work of individuals is incorporated for the benefit of all.

### Keep it simple / Include everything

Most analyses examine some areas of the data in great detail but require only basic summaries of others. We supports this using well-chosen data aggregation, offering multiple levels of detail so that each analyst can decide how far they wish to `zoom in' on each element.

## Conclusion

Some apparently contradictory requirements of data management can be resolved with the above techniques, creating a resource suitable for a wide range of applications without compromise.

